# Longitudinal study on the genetic diversity of extended-spectrum cephalosporin resistant *Escherichia coli* in Dutch veal calves

**DOI:** 10.3389/fmicb.2025.1636304

**Published:** 2025-09-23

**Authors:** T. d. J. Bello Gonzalez, F. Marcato, K. T. Veldman, M. Wolthuis-Fillerup, F. Harders, K. van Reenen, M. S. M. Brouwer

**Affiliations:** ^1^Department of Bacteriology, Host-Pathogen Interaction and Diagnostic Development, Wageningen Bioveterinary Research, part of Wageningen University and Research, Lelystad, Netherlands; ^2^Wageningen Livestock Research, Wageningen University and Research, Wageningen, Netherlands; ^3^Faculty of Veterinary Medicine, Institute for Risk Assessment Sciences (IRAS), Utrecht University, Utrecht, Netherlands

**Keywords:** veal calves, extended-spectrum cephalosporin resistance, *Escherichia coli*, AMR, extended-spectrum beta-lactamase (ESBL), livestock

## Abstract

The present study aimed to evaluate the genetic diversity of Extended Spectrum Cephalosporin resistant (ESC-R) *E. coli* isolates obtained from Dutch veal calves during a longitudinal study, to better understand the genetic background behind the persistent ESC-R *E. coli* colonization in the dairy and veal sectors. Rectal swabs were collected from 683 calves located in 13 Dutch dairy farms 1 day prior to their transportation to 8 veal farms. At the veal farms, rectal swabs were collected at 5 different time points after arrival. A total of 1,202 ESC-R *E. coli* isolates were obtained through selective isolation. Nine out of 13 dairy farms were positive for ESC-R *E. coli* (*n* = 175 isolates), arbitrarily divided in a high prevalence (>50%, *n* = 7) or low prevalence farms (<5%, *n* = 2) In three veal farms, a relatively high frequency of ESC-R *E. coli* was observed (range = 38–84%) while in the remaining five farms, a lower frequency was observed (range = 5–24%). At veal farms, the highest average ESC-R *E. coli* frequency was detected in week two (57.3%). ESBL/AmpC encoding genes were identified by RT-PCR and amplicon sequencing. ESBL genes (*bla*_CTX-M-1_ groups, *bla*_CTX-M-9_ group) and specific point mutations in the promotor region of the chromosomal *bla*_ampC_ gene were identified both at dairy farms and veal farms. A total of 364 ESC-R *E. coli* isolates were further characterized by WGS to determine the genetic relationship using core genome Multi Locus Sequencing Typing (cgMLST). The ESBL-genes *bla*_CTX-M-1_ and *bla*_CTX-M-15_ were predominant, the majority in combination with a non-ESBL *bla*_TEM-1_ gene. In addition, genes encoding resistance against quinolones, aminoglycosides, phenicols, tetracyclines, sulfonamides, and trimethoprim were identified in these isolates. Finally, phylogenetic analysis showed a diverse pool of *E. coli* strains carrying the *bla*_CTX-M-1_ gene, while two genetically closely related sequence types (STs) were identified in *E. coli* strains carrying the *bla*_CTX-M-15_, being ST 4981 and ST 2325, the most predominant STs identified. Our results revealed a population of ESC-R *E. coli* which are genetically similar within veal farms and provides evidence of direct transmission and dissemination of ESC-R *E. coli* between the animals during the rearing process.

## Introduction

In the Netherlands, measures to reduce the antimicrobial use in livestock have been implemented successfully since 2009 (Speksnijder et al., 2015). This has led to a substantial reduction of antimicrobial use in livestock (77.5% in 2023) and the complete ban of the use of third and fourth generation cephalosporins, classified as critically important antibiotics for human health by the World Health Organization (WHO) [Speksnijder et al., 2015; Collignon et al., 2016; Veterinary Medicines Institute (SDa), 2020]. As a result, the prevalence of extended-spectrum beta-lactamase (ESBL) or AmpC-producing *Escherichia coli*, collectively referred here as extended-spectrum cephalosporin resistant (ESC-R)-*E. coli,* decreased or stabilized at a low level in most livestock sectors in the Netherlands between 2014 and 2023 (NETHMAP_MARAN, 2024). The average antimicrobial usage in the veal sector reduced from 29.2 Defined Daily Dose Animal National (DDDA_NAT_) in 2011 to 16.4 DDDA_NAT_ in 2023 (SDa-report, 2023). However, the prevalence of ESC-R *E. coli* in the white veal calf sector unexpectedly increased from 18.7% in 2015 up to 49.5% in 2018, while the prevalence in rosé veal increased from 11.3% in 2015 to 29.3% in 2017. After this period, a slow decrease to 39.9% in 2023 was observed for white veal calves, and 23.9% in rosé veal calves (NETHMAP_MARAN, 2024). The apparent persistence of ESC-R *E. coli* in the veal sector without selective pressure due to the use of 3^rd^ or 4^th^ generation cephalosporins, highlights the need to better understand the dynamics and spread of ESC-R *E. coli* in veal calves, starting from the early life at the dairy farm and during the rearing period at the veal farm. ESC-R *E. coli* are prevalent in veal calves and dairy calves both in Europe and in other large dairy-producing countries such as China, the United States, and Canada (Waade et al., 2021; Masarikova et al., 2025; Kim et al., 2021; Gelalcha et al., 2023; He et al., 2021; de Lagarde et al., 2024).

In a previous manuscript (Bello Gonzalez et al., 2022) we investigated the prevalence of ESC-R *E. coli* obtained in a longitudinal study on white veal calves and we identified whether or not the cumulative effect of antibiotic use at the dairy and veal farms could have contributed to the numbers of ESC-R *E. coli* carriage at veal farm level using statistical regression models. No significant factors could be identified in relation to the prevalence of ESC-R *E. coli*, presumably due to the usage of antimicrobial treatment at herd level on all veal farms included in the study (Bello Gonzalez et al., 2022). To better understand the potential mechanisms and dynamics behind the persistent presence and spread of ESC-R *E. coli* in the dairy and veal calf sector, we performed Whole Genome Sequence (WGS) analysis on a selection of ESC-R *E. coli* isolates obtained at the dairy and veal farms to: (a) identify the genetic similarity of ESC-R producing *E. coli* isolates obtained from calves located at dairy farms; and (b) evaluate the genetic relationship of ESC-R *E. coli* isolates obtained from calves located at veal farms with high and low prevalence of *E. coli.* In addition, we explored the dynamics in ESC-R *E. coli* colonization over time in a subset of animals colonized multiple times during the entire rearing process from two veal farms with high frequency of ESC-R *E. coli.*

## Materials and methods

From March 2019 to May 2020 a longitudinal study was conducted (Bello Gonzalez et al., 2022) during which rectal swabs were collected from 683 calves at the dairy farms of origin (DF; *N* = 13) on the day prior to their transport (age 14 or 28 days) to 8 veal farms (VF). These calves were also sampled at the veal farms at 5 timepoints (week 2, 6, 10, 18, and 24 after arrival). Through selective isolation on MacConkey agar supplemented with cefotaxime (1 mg/L), a total of 1,202 ESC-R *E. coli* isolates were identified over time from rectal swab samples. See Bello Gonzalez et al. (2022) for the complete study design and identification of ESC-R *E. coli* isolates.

### Characterization of ESBL/AmpC resistance genes by RT-PCR

Cell lysates obtained from the 1202 ESC-R *E. coli* isolates were screened for the presence of ESBL/AmpC resistance encoding genes by a multiplex Real-time PCR assay on a light cycler System (Applied Biosystems, 7500 Fast Real-Time PCR System). The multiplex-Real-time PCR assay included the detection of the ESBL genes: *bla*_CTX-M-1_ group, *bla*_CMY_, *bla*_TEM_, and *bla*_SHV_ as previously described (Geurts et al., 2017). Isolates that were negative for these genes were subjected to a second round of PCR screening for the ESBL/AmpC genes: *bla*_CTX-M-2_ group, *bla*_CTX-M-8/25_, *bla*_CTX-M-9_ group and chromosomal *bla*_ampC_ as described previously by Dierikx et al. (2012) and Liakopoulos et al. (2016). After the initial screening, a confirmation of the detected ESBL/AmpC gene was performed by DNA Sanger sequencing (3,130 Genetic Analyzer) as previously described (Liakopoulos et al., 2016). The nucleotide sequences were compared with reference sequences obtained from GenBank using the Sequencher 5.4.6 software (Gene Codes Corporation, Michigan, United States) for the identification of the specific gene variants present in the isolates.

### Whole genome sequencing analysis

To characterize the genetic diversity of these isolates, we performed WGS of a subset of ESC-R *E. coli* isolates. For the selection of the isolates, we have included: (a) A total of 111 ESC-R *E. coli* isolates obtained from four out of seven dairy farms with high frequency of ESC-R *E. coli* (>50%), (b) A total of 253 ESC-R *E. coli* isolates obtained from two veal farms with the highest frequency of ESC-R *E. coli* (VF 6 (83%) and VF 7 (61%), *n* = 166) and 87 ESC-R *E. coli* from two veal farms with a low frequency of ESC-R *E. coli* (VF 1 (23%) and VF 8 (17%), *n* = 87). Bacterial DNA was isolated and purified with the Qiagen Blood and tissue DNA isolation kit (Qiagen). The DNA concentration was measured using the CLARIOstar Plus (BMG Labtech). The library construction was performed using the KAPA HyperPlus Kit (KAPA BIOSYSTEMS). DNA samples were sequenced based on 250-300-bp paired read length on the Illumina Miseq sequencer using the MiSeq Reagent kit v3 (Illumina). Sequence analysis was performed as previously described (Brouwer et al., 2023). Quality was assessed before and after read polishing with FastQC v0.12.1. Polishing was performed with tools of the BBduk suite v39.06, including filtering for PHI-X sequences, error correction, sequence adaptor trimming, quality trimming to Q = 30, and normalizing the data to 100x coverage. Assembly was performed using Spades-based Shovill, v1.1.0. Assembly quality was assessed using QUAST v5.3.0, assemblies with > 500 contigs or assembly size < 4 Mb or > 6 Mb were rejected. Assembly statistics are included in Supplementary Table S5. AMR-genes were detected using ResFinder v4.1.0, with a minimum of 90% sequence coverage and 90% sequence identity. Analysis of genomic clusters using cgMLST was performed using Ridom Seqsphere+ (Ridom GmbH, Münster, Germany), which considers each non-identical allele, which may include one or multiple SNPs, or small indels per gene, as a single allelic difference. Isolates with 10 allelic differences or less were considered a cluster. For each cluster, annotation of the genomes was performed by Prokka v1.12. ROARY v3.12.0 was used to determine a core-gene alignment for the genomes within the cluster. A pairwise comparison of the genomes in the cluster was performed with snp-dists v0.8.2 to determine the range of SNPs that was detected within the cluster.

### Accession number

The WGS datasets generated in this study have been deposited in the European Nucleotide Archive (ENA) at EMBL-EBI under accession number PRJEB83274.

## Results

The results of selective culturing of ESC-R *E. coli* at the dairy farms and veal farms were previously reported (Waade et al., 2021). In summary, the calves from four dairy farms were culture negative for ESC-R *E. coli* (DF1, DF3, DF5 and DF6), while the remaining nine farms can arbitrarily be divided in two prevalence farms, low prevalence (DF8 2.0%, DF12 3.6%) and seven farms with an average of detected ESC-R *E. coli* at or above the median of all farms (19.0%) which were considered high prevalence (DF2 86.0%, DF4 71.1%, DF7 64.0%, DF9 17.6%, DF10 39.5%, DF11 46.6% and DF13 25%).

For the veal farms, the median prevalence was 23.6%, of which four farms were below (VF1 23.4%, VF4 4.8%, VF5 14.8%, VF8 17.3%) and four farms above (VF2 37.0%, VF3 23.9%, VF6 83.0%, VF7 61.6%).

### Frequency and distribution of ESBL/AmpC resistance genes present at dairy and veal farms detected by RT-PCR

**Figure 1:**
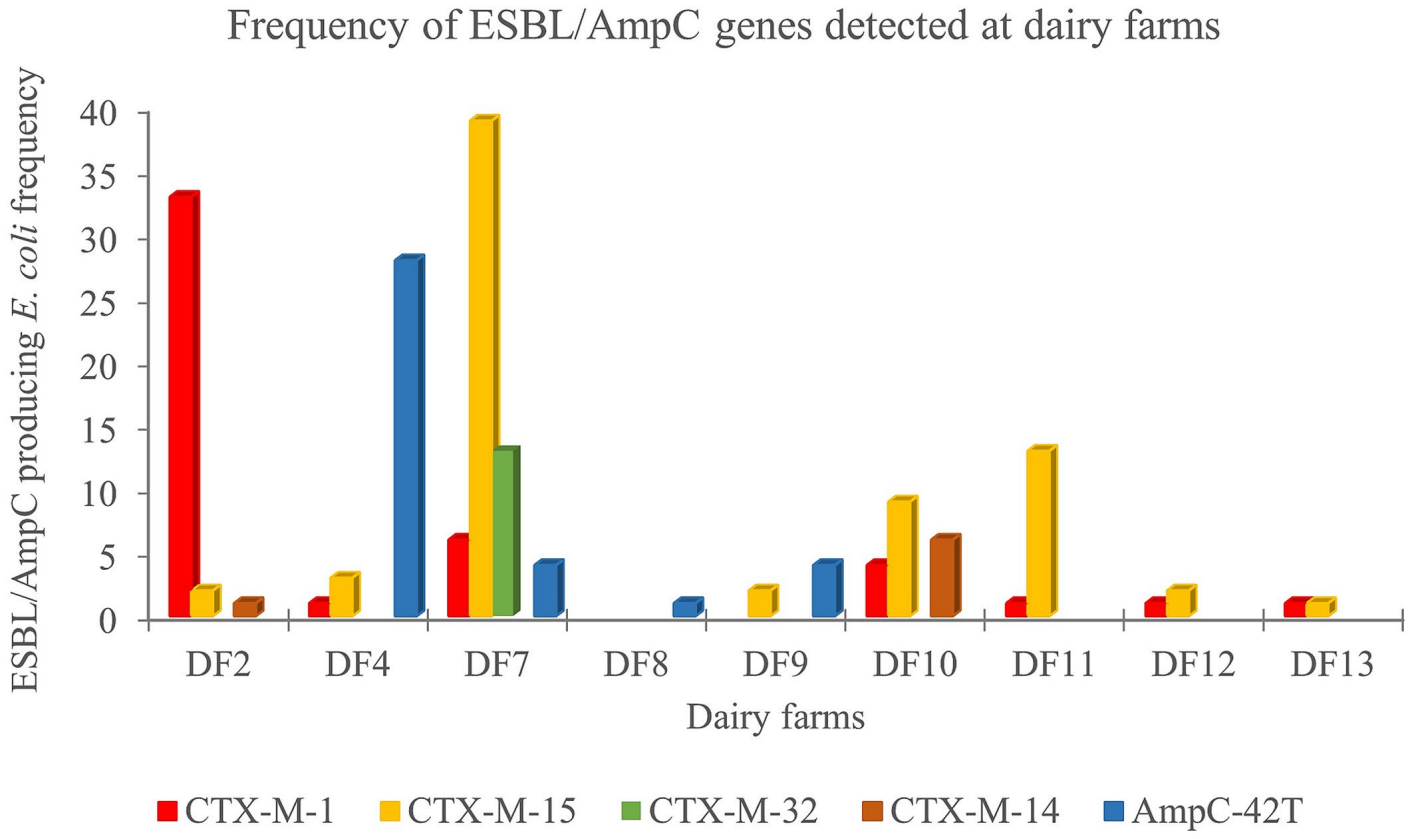
Frequency and distribution of ESBL/AmpC resistance genes in rectal swabs samples collected at 13 dairy farms (DF).

At the dairy farms, the ESBL and/or AmpC encoding genes were identified in 175 ESC-R *E. coli* from nine farms on the day before transportation to the veal farms. Differences in frequency and distribution of ESBL/AmpC resistance genes were observed between dairy farms (Figure 1). From the nine dairy farms positive for ESC-R *E. coli*, seven were considered high-prevalence farms (>19.0%), in which a mixture of ESBL/AmpC genes were detected, although a dominant gene was detected in all farms. DF2 was dominated by *bla*_CTX-M-1_ (91.6%, *n* = 33/36), while in DF7, DF10 and DF11, the *bla*_CTX-M-15_ gene was the predominant gene (62.9% *n* = 39/62; 47.3% *n* = 9/19; 92.8% *n* = 13/14). Other genes detected with low frequency in these dairy farms correspond to *bla*_CTX-M-14_ (DF2 2.7% *n* = 1/36; DF10 31.5% *n* = 6/19), and *bla*_CTX-M-32_ which was detected only in DF7 (20.9% *n* = 13/62). Finally, point mutations in the promotor region of chromosomal AmpC (C-42 T) were predominantly present in ESC-R *E. coli* isolates from DF4 (97%, *n* = 31/32) and DF9 (66.6% *n* = 4/6). This specific promoter mutation is associated with resistance to cephalosporins and other beta-lactam antibiotics (Peter-Getzlaff et al., 2011; Bortolaia et al., 2020). In the remaining two low prevalence farms (<19.0%), *bla*_CTX-M-15_, and *bla*_CTX-M-1_, were identified in DF12, while in DF8 only a point mutation in the promotor region of chromosomal AmpC (C-42 T) mutation was detected.

**Figure 2:**
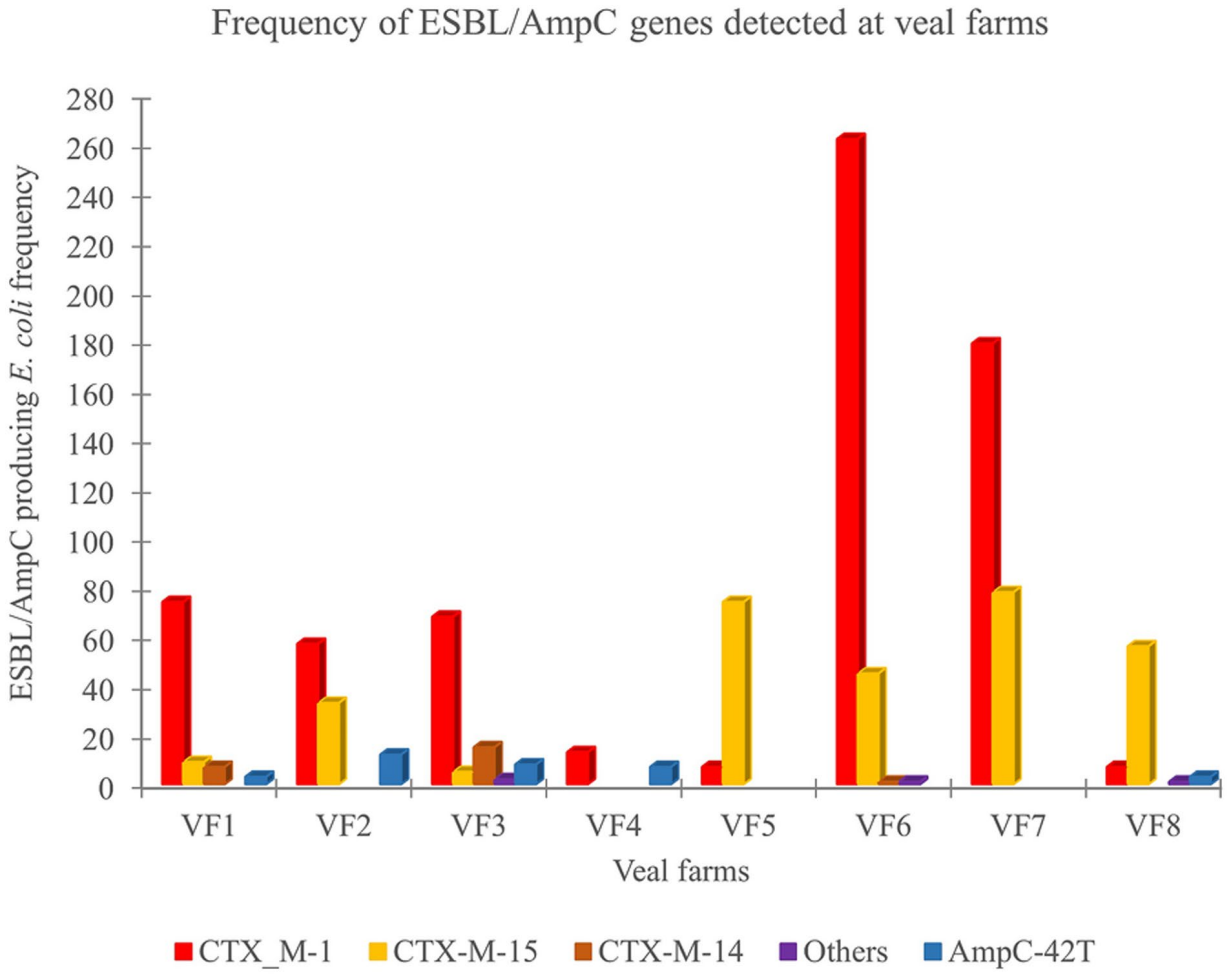
Frequency and distribution of ESBL/AmpC resistance genes in rectal swabs collected in 8 veal farms (VF).

The overall frequency of ESBL and/or AmpC encoding genes was identified in 1027 ESC-R *E. coli* isolates obtained throughout the rearing phase in eight veal farms. Differences in the frequency and distribution of the ESBL/AmpC encoding genes were observed between veal farms (Figure 2). In the veal farms with high frequencies of ESC-R *E. coli* (23.6%) were observed in VF2, VF3, VF6 and VF7, in which *bla*_CTX-M-1_ (*n* = 565, 73.7%) and *bla*_CTX-M-15_ (*n* = 161, 21%) genes were the most predominant ESBL genotype detected. In VF3 and VF6 the *bla*_CTX-M-14_ gene was detected (*n* = 16, 3.9%), while *bla*_CTX-M-32_ gene was only detected in VF6. Gene *bla*_CMY-2_ (*n* = 1, 1.0%) and *bla*_TEM-52c_ (*n* = 1, 1.0%) were detected only in VF3. In VF2 and VF3 isolates with point mutations in the promotor region of chromosomal AmpC (C-42 T) (*n* = 20, 10%) were detected. In the other four veal farms (VF1, VF4, VF5, and VF8), low frequency of ESC-R *E. coli* (<23.6%) was detected, in which the most prevalent ESBL genes were also *bla*_CTX-M-1_ (*n* = 101, 38.6%), while *bla*_CTX-M-15_ (*n* = 139, 57.6%) was detected only in VF1, VF5, and VF8. In addition, the presence of *bla*_CTX-M-14_ (*n* = 7, 7.5%) was only detected in VF1. Moreover, *bla*_CTX-M-65_ was found in a single isolate from VF8. In VF1 and VF8 point mutation in the promotor region of chromosomal AmpC (C-42 T) (*n* = 6, 3.7%) was identified.

### Whole genome sequencing analysis on the genetic similarity of ESC-R *Escherichia coli* isolates at the dairy farms

In order to evaluate the diversity and genetic similarity of ESC-R *E. coli* isolates obtained from calves at dairy farms, we have selected a subset of 111 ESC-R *E. coli* isolates from four out of seven high-frequency dairy farms (DF2, *n* = 33/36; DF7, *n* = 60/62; DF10, *n* = 10/19; and DF11, *n* = 8/14) for WGS analysis.

**Figure 3:**
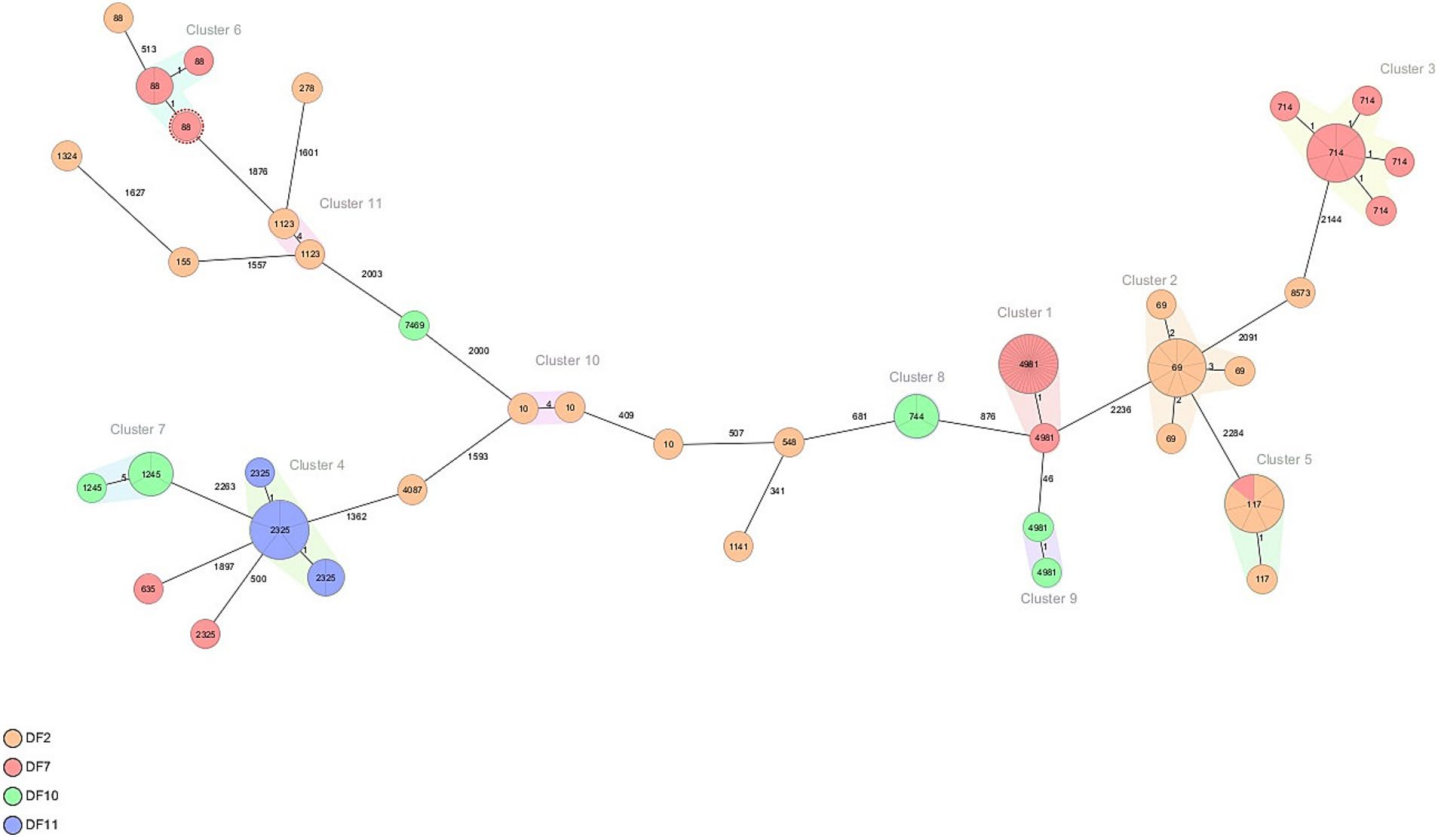
Minimum spanning tree of ESC-R *Escherichia coli* isolated in four out of 13 Dutch dairy farms (DF2, DF7, DF10 and DF11). Details are shown in Table 1.

Over the four farms, 98 of the 111 isolates represented a cluster that was detected more than once on a farm. On DF2, four clusters were detected of 2–12 isolates (see Table 1; Figure 3). These four clusters represented four MLST types (ST10, ST69, ST117, ST1123) all of which contained *bla*_CTX-M-1_. On DF7, three clusters were detected, Cluster 1 contained 42 isolates of ST4981 with *bla*_CTX-M-15_, which was the most dominant ESBL detected in this farm, Cluster 3 contained 11 isolates of ST714 containing *bla*_CTX-M-32_ and Cluster 6 contained four isolates of ST88 with the AmpC (C-42 T) promotor mutation. On DF10, three smaller clusters were detected, Cluster 7 contained four isolates of ST1245 with *bla*_CTX-M-14_; Cluster 8 contained three isolates of ST744 with *bla*_CTX-M-15,_ and Cluster 9 contained 2 isolates of ST4981 with *bla*_CTX-M-15_. On DF11 Cluster 4 was detected of 8 isolates ST 2325 with *bla*_CTX-M-15_. Notably, the *E. coli* isolates carrying *bla*_CTX-M-15_ belonging to ST4981 were circulating on DF 7 and DF 10 in the same period of time, corresponding to August–November 2019. However, genetic distance analysis indicates that these isolates belong to two different clusters. Pairwise comparison of the SNPs between isolates in the different clusters was used to confirm the genetic relationship of the isolates. While Cluster 7 and 11 contain more diverse isolates, Cluster 1, 3, 5, 6, 9 and 10 consist of closely related isolates, while Cluster 2, 4 and 8 contain both highly related and some less related isolates.

**Table 1:** ESC-R *Escherichia coli* clusters detected at dairy farms the day prior to transport (age of calves: 14 or 28 days old).

Cluster	# isolates	Farm	Months	ST	ESBL gene	SNPs
Cluster 1	42	DF7	Aug, Sep, Oct, Nov	4,981	*bla*_CTX-M-15_	0–23 (0)
Cluster 2	12	DF2	Apr, May, Jun, Aug, Oct, Nov	69	*bla*_CTX-M-1_	0–92 (26)
Cluster 3	11	DF7	Apr, Jul, Aug	714	*bla*_CTX-M-32_	0–32 (1)
Cluster 4	8	DF11	Aug, Sep, Oct, Nov	2,325	*bla*_CTX-M-15_	0–140 (1)
Cluster 5	8	DF2, DF7	Apr, Jun, Aug, Sep, Oct	117	*bla*_CTX-M-1_	0–23 (2)
Cluster 6	4	DF7	Apr, Jun, Jul	88	AmpC (C-42 T)	10–34 (20)
Cluster 7	4	DF10	Jun, Aug	1,245	*bla*_CTX-M-14_	84–364 (224)
Cluster 8	3	DF10	Sep, Oct	744	*bla*_CTX-M-15_	1–115 (1)
Cluster 9	2	DF10	Oct, Nov	4,981	*bla*_CTX-M-15_	5
Cluster 10	2	DF2	Jul	10	*bla*_CTX-M-1_	11
Cluster 11	2	DF2	Apr, Oct	1,123	*bla*_CTX-M-1_	1,357

Per cluster, the range of SNPs is indicated with the median number of SNPs included in parentheses.

Analysis of the predicted resistance patterns based on WGS data shows that 88 of 111 ESC-R *E. coli* isolates were multidrug resistant. Genes encoding resistance to aminoglycosides (*n* = 88), florfenicol (*n* = 16), sulfonamides (*n* = 67), quinolones (*n* = 58), tetracycline (*n* = 77), trimethoprim (*n* = 68) and chloramphenicol (*n* = 11) (Supplementary Table S1) were detected.

### Whole genome sequencing analysis on the genetic similarity of ESC-R *Escherichia coli* isolates at the veal farms

From eight veal farms in the study, two farms were selected with an average prevalence above the median prevalence (>23.5%) for WGS analysis; 90 out of a total of 309 ESC-R *E. coli* isolates from VF6 and 76 out of 257 isolates from VF7. Furthermore, two farms below the median prevalence were studied; 26 of 93 ESC-R *E. coli* isolates from VF1 and 61 out of 67 isolates from VF8.

**Figure 4:**
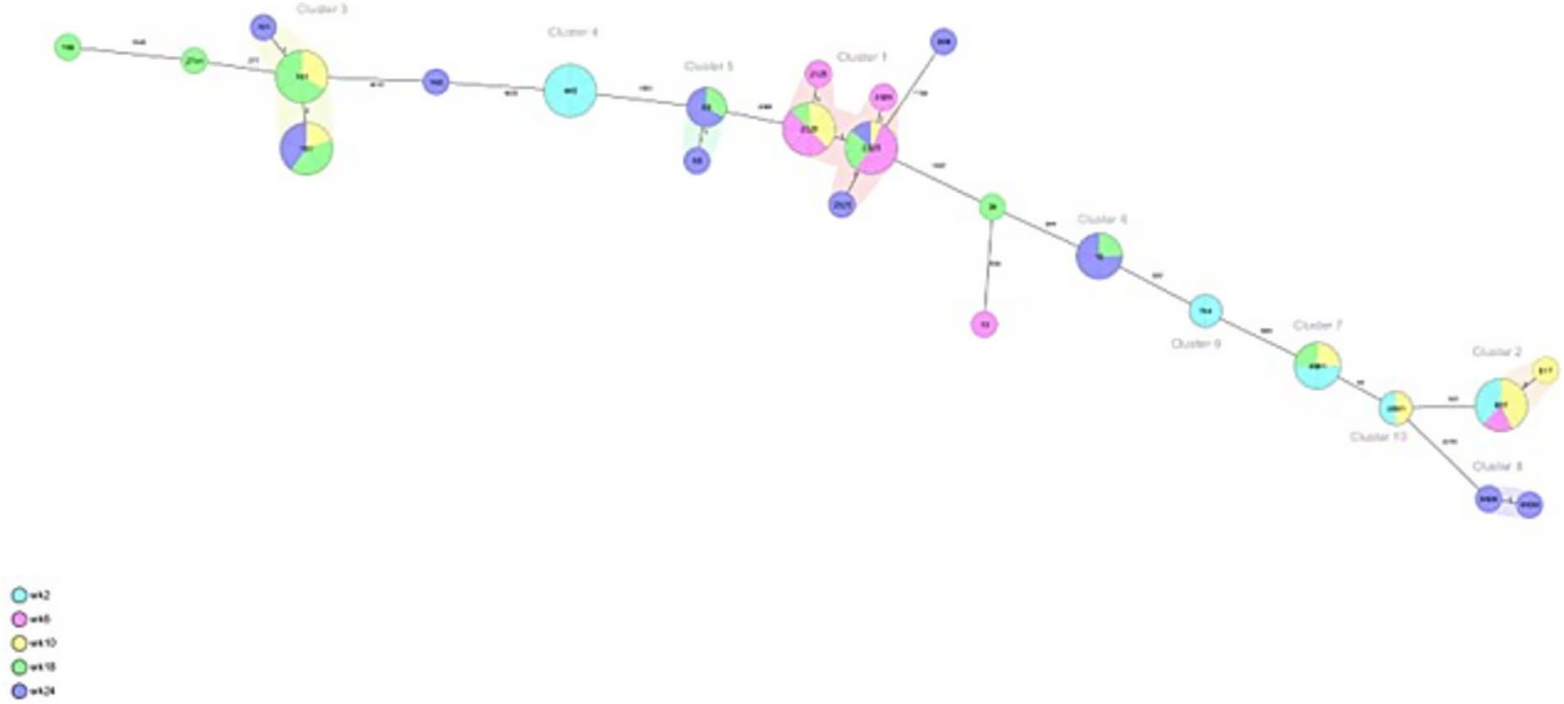
Minimum spanning tree of ESC-R *Escherichia coli* isolated at VF6 at different timepoints (week 2, 6, 10, 18 and 24 post-transport). Details are shown in Table 2.

**Figure 5:**
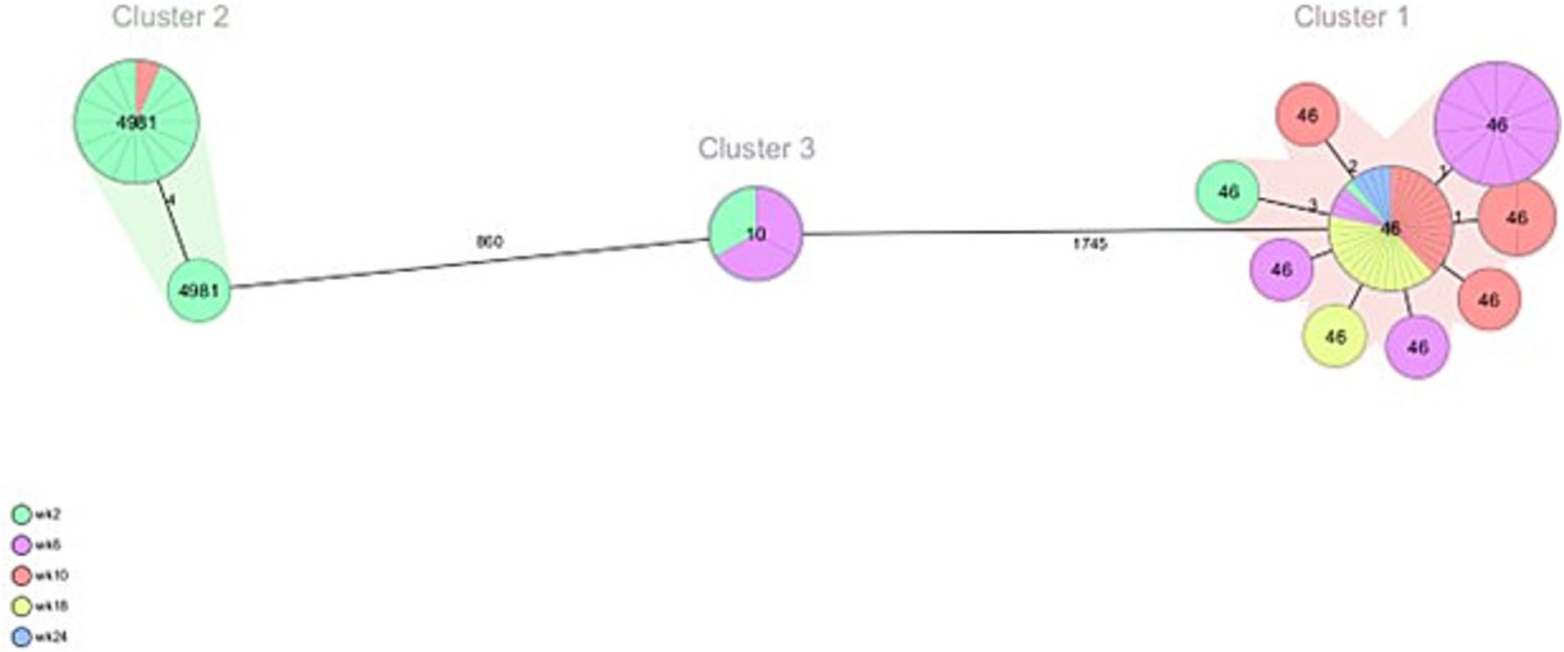
Minimum spanning tree of ESC-R *Escherichia coli* isolated at VF7 at different timepoints (week 2, 6, 10, 18 and 24 post-transport). Details are shown in Table 3.

From veal farms with a high frequency of ESC-R *E. coli*, a total of 160 out of 166 isolates were part of clusters that were detected multiple times per farm. In VF6, nine clusters were detected of 2–26 isolates per cluster (Figure 4; Table 2). Five clusters of isolates encoding *bla*_CTX-M-15_ belonged to ST10, ST88, ST617, ST744, and ST4981. Two of these clusters were also detected on VF7 (ST10 *n* = 3, ST4981 *n* = 17) (Figure 5; Table 3). On VF6, a circulating pattern of STs was observed over time, carrying *bla*_CTX-M-15_, of which ST617 and ST4981 were detected after arrival at the veal farm (week 2) and dominated in week 6 and 10, after which ST10 and ST88 persisted in week 18 and week 24. In the group of isolates carrying *bla*_CTX-M-1_ in this VF, four clusters belonged to ST2325, ST101, ST5926, and ST448 were identified, of which ST448 was only present in week 2 while ST101 and ST2325 were circulating during the rest of the fattening process until slaughter. The ST5926 was only detected in week 24. Of the 10 detected clusters on VF6, All clusters consist of related to highly related *E. coli* isolates with 0 to 31 SNPs, except for Cluster 9, consisting of 2 isolates with 465 SNPs difference. In VF7, one additional cluster was detected, which contained 56 isolates of ST46 carrying *bla*_CTX-M-15_, which were all confirmed to be highly related with 0–6 SNPs between them. Cluster 2 and 3 both contain some related and some more divergent isolates.

**Table 2:** ESC-R *Escherichia coli* clusters detected at veal farm 6 at different timepoints (week 2, 6, 10, 18 and 24 post-transport).

Cluster	# isolates	Week	ST	ESBL gene	SNPs
Cluster 1	26	6, 10, 18, 24	2,325	*bla*_CTX-M-1_	0–27 (0)
Cluster 2	22	2, 6, 10	617	*bla*_CTX-M-15_	0–3 (0)
Cluster 3	12	10, 18, 24	101	*bla*_CTX-M-1_	0–3 (0)
Cluster 4	6	2	448	*bla*_CTX-M-1_	0–1 (0)
Cluster 5	4	18, 24	88	*bla*_CTX-M-15_	2–16 (2)
Cluster 6	4	18, 24	10	*bla*_CTX-M-15_	4–13 (5)
Cluster 7	4	2, 10, 18	4,981	*bla*_CTX-M-1_, *bla*_CTX-M-15_	0–31 (1)
Cluster 8	2	24	5,926	*bla*_CTX-M-1_	5
Cluster 9	2	2	744	*bla*_CTX-M-15_	465
Cluster 10	2	2, 10	4,981	*bla*_CTX-M-15_	0

Per cluster, the range of SNPs is indicated with the median number of SNPs included in parentheses.

**Table 3:** ESC-R *Escherichia coli* clusters detected at veal farm 7 at different timepoints (week 2, 6, 10, 18 and 24 post-transport).

Cluster	# isolates	Week	ST	ESBL gene	SNPs
Cluster 1	56	2, 6, 10, 18, 24	46	*bla*_CTX-M-15_	0–6 (2)
Cluster 2	17	2, 10	4,981	*bla*_CTX-M-15_	0–123 (0)
Cluster 3	3	2, 6	10	*bla*_CTX-M-15_	13–75 (13)

Per cluster, the range of SNPs is indicated with the median number of SNPs included in parentheses.

In 2 veal farms with low frequency of ESC-R *E. coli*, VF1 and VF8, a total of 75 out of 87 *E. coli* isolates represented clusters that were detected multiple times in a veal farm (Supplementary Tables S2, S3). In VF1, three clusters were detected all carrying *bla*_CTX-M-1_. Cluster 1 consisted of six ST648 isolates, Cluster 2 contained nine ST69 isolates, and Cluster 3 contained three ST117 isolates. The majority of these clusters were detected during the first weeks (week 2 and 6) after arrival of calves at the veal farm. While the isolates in Cluster 2 are highly related with 4 out of 5 isolates containing 0 or 1 SNPs, the isolates in Cluster 1 are more distantly related, and the isolates in Cluster 3 are not related. In VF8, isolates carrying *bla*_CTX-M-15_ were represented by 4 clusters which belong to ST46, ST2325, and ST4981. These clusters were also present in the first weeks after arrival of calves at the veal farm. In this VF, one additional cluster was identified which consisted three ST5229 isolates carrying *bla*_CTX-M-1_. All 5 clusters consist of highly related *E. coli* with 0 to 18 SNPs between the isolates within the clusters.

In 25 *E. coli* isolates carrying *bla*_CTX-M-15_ gene from VF6 (ST617) and VF7 (ST10), the presence of *bla*_OXA-1_ was detected. The *mcr*-1 gene responsible for colistin resistance was identified in seven *E. coli* isolates carrying *bla*_CTX-M-1_ all obtained from VF6 (*n* = 6, ST448 and *n* = 1, ST10). Moreover, the *lnu*(F) gene conferring resistance to lincosamides was identified in 31 *E. coli* isolates originating from VF1 (*n* = 6) and VF8 (*n* = 3) carrying *bla*_CTX-M-1_, and from VF6 (*n* = 22) carrying *bla*_CTX-M-15_. Moreover, in 240 ESC-R *E. coli* isolates genes conferring to aminoglycoside (*n* = 240), florfenicol (*n* = 170), sulfonamides (*n* = 220), tetracycline (*n* = 240), quinolones (*n* = 222), trimethoprim (*n* = 195) and chloramphenicol (*n* = 138) (Supplementary Table S2) were detected.

In addition, the diversity and dynamics of ESC-R *E. coli* colonization over time was explored within animals colonized multiple times during the entire rearing process from the two veal farms with the highest frequency [VF 6 (*n* = 20 animals, 100 isolates) and VF 7 (*n* = 19 animals, 82 isolates)], including the sampling moment at the dairy farms. An overview per animal is shown in Supplementary Table S4, in which the clusters are shown for which the ESC-R *E. coli* belong to. The most dominant ESBL genes detected in calves from VF6 were *bla*_CTX-M-1_ (*n* = 51 isolates) and *bla*_CTX-M-15_ (*n* = 47 isolates). In 95% of the animals a dynamic change in ESBL gene carriage between the two most dominant ESBL genes was detected. The presence of the *bla*_CTX-M-15_ gene was detected in the earlier samples collected 1 day prior to transportation to the veal farm or after week 2 at the veal farm. After week 2 at the veal farm, a change in colonization was observed where the *bla*_CTX-M-1_ gene was the dominant ESBL gene present during the rearing period until week 18 when the presence of *bla*_CTX-M-15_ gene was observed again. In the group of isolates carrying the *bla*_CTX-M-15_ gene, ST617 of Cluster 2 (*n* = 22) was detected in 7 animals at least two times between week 2 and 10 at the veal farm. The second most dominant clone belonged to ST4981 (n = 10) and was detected in 2 animals at least two times, both coming from dairy farms with high frequency of ESC-R *E. coli* (DF 6 and DF 7). From the group of isolates carrying *bla*_CTX-M-1_, ST2325 (n = 26) was detected in eight animals two (*n* = 6) to three (*n* = 2) times between week 6 and 24. The second most dominant ST carrying *bla*_CTX-M-1_ belonging to ST101 (*n* = 12) was detected in three animals at least two times, two of them coming from DF 7. Overall, these 20 animals had an average of 5 sampling moments on which they were colonized by ESC-R *E. coli*, and on average these were approximately 4 different *E. coli* lineages per animal. In VF 7, the dominant ESBL gene was *bla*_CTX-M-15_, present in 99% of the isolates with two predominant ST-types. ST46 (n = 56) was detected in 18 animals at least two to four times over the entire study period, mainly during week 6 and 24, and ST4981 (*n* = 22) was detected in 4 animals at least two times between week 0 and week 2 at the veal farm. Remarkably, a reoccurring pattern of ESBL gene carriage was observed in 14 animals, all of them being colonized between week 0 and 2 with ST4981 and between week 6 to 24 with ST46.

One veal calf in VF7 was only colonized by the same ST46 *E. coli* from Cluster 1 over time (*n* = 3 isolates). Other animals in this VF were also colonized by other ESC-R *E. coli*, but the same ST46 *E. coli* of Cluster 1 was also detected up to 4 times in the same animal. Overall, the 19 animals that were analyzed on VF7 had an average of 4.4 sampling moments on which they were colonized by ESC-R *E. coli*, with an average of 2.5 different *E. coli* lineages per animal, emphasizing the lower diversity of ESC-R *E. coli* detected on this farm.

## Discussion

The aim of this study was to evaluate the dynamics and genetic diversity of ESC-R *E. coli* isolates obtained during a longitudinal study on Dutch veal calves, to establish the genetic background of *E. coli* colonization dynamics present in veal calves during the entire rearing process from the dairy farm of origin to the veal farms, until slaughter age. In our study we observed a distinction between farms based on ESC-R *E. coli* colonization frequency over time.

Here we have used the median frequency of ESC-R *E. coli* colonization as an arbitrary cut-off to distinguish low and high-prevalence farms. For the dairy farms, this resulted in a low prevalence farms ranging from 0 to 3.5% and high prevalence farms ranging 18.9 to 86%. In the veal farms, using the median of 23.6 resulted in a low range of 4.8 to 23.6 and the high range of 23.9 to 83.0%. Although the difference between the range of low and high-prevalence farms is very close together, this cut-off was only used for the selection of farms for WGS analysis and descriptive purposes.

In our previous publication from this study (Bello Gonzalez et al., 2022), we reported that the administration of individual antibiotic treatment applied before week 2 and 6 upon arrival to the veal farm might be the explanation for the increase in ESC-R *E. coli* colonization frequency. Previous studies have reported that exposure of young calves to antibiotics via colostrum and feeding practices may play a role in the increased colonization in young calves (Tetens et al., 2019; Dupouy et al., 2021). In addition, other factors such as housing type, the environment and management practices at the dairy farm have been associated with the increase of ESC-R *E. coli* colonization over time (Pereira et al., 2014). Moreover, Gonggrijp et al. (2023) have recently shown that a significant increase on gut colonization with ESC-R *E. coli* in young calves up to 21 days of age occurs only short-term, followed by a decrease on ESBL/AmpC *E. coli* gut colonization on calves followed up to 12 months at dairy farms (Gonggrijp et al., 2023). In our study, no statistical significant differences in frequency of ESC-R *E. coli* were detected in calves the day prior to transport (14 or 28 days of age) (Waade et al., 2021).

Along with the differences in frequency, we also observed a difference in the ESC-R *E. coli* genes detected. In dairy and veal farms, the family of *bla*_CTX-M_ genes, mainly *bla*_CTX-M-1_ and *bla*_CTX-M-15,_ were predominant, followed by the AmpC (C-42 T) point mutation in the promotor region associated with ESC resistance (Peter-Getzlaff et al., 2011; Bortolaia et al., 2020). Similar results were previously reported in the Netherlands by Gonggrijp et al. (2016) and Hordijk et al. (2019). The presence of these genes have also been identified in the colostrum of newborn dairy calves (He et al., 2021) suggesting that feeding colostrum can serve as a possible transmission vector of ESC-R *E. coli*. In our study, the frequency of ESC-R *E. coli* colonization decreased over time (up to week 24), as previously described by Hordijk et al. (2013). The variety of *bla*_CTX-M_ genes reported in our study indicate diverse pathways for the dissemination of these resistance genes in the calves. These genes have been reported in isolates obtained from humans, food-producing animals including cattle, poultry, pigs, and from companion animals (Ewers et al., 2012; Bortolami et al., 2019).

Moreover, in our study, potentially clonally related ECR-*E. coli* STs were detected. While clusters were defined as <10 allelic differences by cgMLST, highly related isolates are defined as < 10 SNPs in a pairwise comparison, and related isolates is used for isolates with <40 SNPs in a pairwaise comparison (Muloi et al., 2022; Roer et al., 2018). At dairy farms, ST4981 and ST2325 containing *bla*_CTX-M-15_, ST69 and ST117 containing *bla*_CTX-M-1_ and ST88 with the AmpC (C-42 T) point mutation were the predominant STs detected. The ECR-*E. coli* ST69, ST117, and ST88 encoding *bla*_CTX-M-1_, *bla*_CTX-M-15_ and other ESBL-genes have been previously reported in cattle and in human isolates from the Netherlands, Germany, and United Kingdom (Day et al., 2016; Day et al., 2019). At veal farms, ST2325, ST46, ST4981, and ST617 containing *bla*_CTX-M-15_ circulated mainly from week 2 up to week 10 while ST46, ST88, and ST10 circulated during the entire period of the study; likewise, ST69, ST117 containing *bla*_CTX-M-1_ appeared in the first weeks after arrival to the veal farms. On the contrary, ST2325 and ST101 were only present in the latest period of the rearing process until slaughter. The STs detected in our study have globally spread in animals and humans (Ewers et al., 2012; Wu et al., 2013). Likewise, ST2325 one of the two predominant STs circulating at the dairy farm and veal farms, has previously been identified in isolates obtained from raw milk in Germany carrying *bla*_CTX-M-15_ (Irrgang et al., 2017) and a human carrying *bla*_CTX-M-14_ and *bla*_CTX-M-55_ from Thailand (Seenama et al., 2019). Moreover, ST4981 the second predominant ST circulating at the dairy and veal farm has previously been found in ESBL-*E. coli* isolates obtained from clinical samples from the Middle East and Asia (Yasir et al., 2020).

The in-depth genetic analysis of the ESC-R *E. coli* in this study confirms that some of the ESC-R clones isolated at the veal farms originated at the dairy farms. However, the high diversity of clones detected at the veal farms is most likely a consequence of the Dutch husbandry system, where animals arriving to the veal farm originate from several dairy farms located across the country as well as from animals imported from other European countries. Our results confirm the complexity of ESC-R *E. coli* dynamics on veal farms with the temporality of predominant clones in addition to the comings and goings of ESC-R variants in the gut of individual animals. Whole genome sequencing analysis of a subset of ESC-R *E. coli* isolates from dairy and veal farms revealed the co-occurrence of genes encoding resistance against quinolones, aminoglycosides, phenicols, tetracyclines, sulfonamides, trimethoprim. These resistance patterns have been previously observed in *E. coli* isolates from dairy calves and adult dairy cattle (Taylor et al., 2021; Waade et al., 2021). In our previous study (Bello Gonzalez et al., 2022), the use of antibiotics at dairy farms (individual treatment) and at veal farms (individual and batch treatment) may have an impact on the repertoire of resistance genes circulating in the calves during the entire rearing process, which could contribute to the co-selection of multi-drug resistant ESC-R *E. coli* isolates, in this particular case dominated by the *bla*_CTX-M_ gene family. Our study highlights the additional value of performing longitudinal studies to understand the relative abundance of ESC-R *E. coli* in veal calves.

## Data Availability

The datasets presented in this study can be found in online repositories. The names of the repository/repositories and accession number(s) can be found in the Materials and methods.
